# Cathelicidin Contributes to the Restriction of *Leishmania* in Human Host Macrophages

**DOI:** 10.3389/fimmu.2019.02697

**Published:** 2019-11-22

**Authors:** Peter Crauwels, Elena Bank, Bianca Walber, Ulf Alexander Wenzel, Birgitta Agerberth, Menberework Chanyalew, Markos Abebe, Renate König, Uwe Ritter, Norbert Reiling, Ger van Zandbergen

**Affiliations:** ^1^Division of Immunology, Paul-Ehrlich-Institute, Federal Institute for Vaccines and Biomedicines, Langen, Germany; ^2^Institute for Microbiology and Biotechnology, University of Ulm, Ulm, Germany; ^3^Institute for Medical Microbiology and Hygiene, University Clinic of Ulm, Ulm, Germany; ^4^Department of Microbiology and Immunology, Mucosal Immunobiology and Vaccine Center (MIVAC), Institute of Biomedicine at Sahlgrenska Academy, University of Gothenburg, Gothenburg, Sweden; ^5^Division of Clinical Microbiology, Department of Laboratory Medicine, Karolinska University Hospital, Karolinska Institutet, Stockholm, Sweden; ^6^Research and Innovation Directorate, Armauer Hansen Research Institute (AHRI), Addis Ababa, Ethiopia; ^7^Research Group “Host-Pathogen Interactions”, Paul-Ehrlich-Institute, Federal Institute for Vaccines and Biomedicines, Langen, Germany; ^8^Regensburg Center for Interventional Immunology (RCI), Institute of Immunology, University Medical Center Regensburg and University of Regensburg, Regensburg, Germany; ^9^Division of Microbial Interface Biology, Research Center Borstel, Leibniz Center for Medicine and Biosciences, Borstel, Germany; ^10^Institute of Immunology, Johannes Gutenberg University, Mainz, Germany; ^11^Research Center for Immunotherapy (FZI), University Medical Center, Johannes Gutenberg-University Mainz, Mainz, Germany

**Keywords:** *Leishmania*, human macrophages, vitamin D, cathelicidin (LL-37), human primary immune cells, antimicrobial activity

## Abstract

In cutaneous Leishmaniasis the parasitic control in human host macrophages is still poorly understood. We found an increased expression of the human cathelicidin *CAMP* in skin lesions of Ethiopian patients with cutaneous leishmaniasis. Vitamin D driven, Cathelicidin-type antimicrobial peptides (CAMP) play an important role in the elimination of invading microorganisms. Recombinant cathelicidin was able to induce cell-death characteristics in *Leishmania* in a dose dependent manner. Using human primary macrophages, we demonstrated pro-inflammatory macrophages (hMDM1) to express a higher level of human cathelicidin, both on gene and protein level, compared to anti-inflammatory macrophages (hMDM2). Activating the CAMP pathway using Vitamin D in hMDM1 resulted in a cathelicidin-mediated-*Leishmania* restriction. Finally, a reduction of cathelicidin in hMDM1, using a RNA interference (RNAi) approach, increased *Leishmania* parasite survival. In all, these data show the human cathelicidin to contribute to the innate immune response against Leishmaniasis in a human primary cell model.

## Introduction

The disease Leishmaniasis is still affecting 12 million people worldwide, of which up to 30,000 cases die yearly (1, 2). Up to date, no vaccine is available and treatment is not always evident due to the socioeconomic conditions in the affected countries (3, 4). Our knowledge regarding the interaction of *Leishmania* with its human host cell, the macrophage, is still fragmentary, as little is known with respect to antimicrobial mechanisms restricting *Leishmania* growth in human primary macrophages. Moreover, few data is available demonstrating which macrophage phenotype is the most superior for *Leishmania* survival or killing. The human body comprises a broad spectrum of different macrophage phenotypes, related to distinct functional properties (5). Herein, the M1/M2 polarization has been the main framework for years in the field of immunology. In the murine system, “alternatively activated” type 2 macrophages are shown to support *Leishmania* parasite replication and persistence via an increased arginase I activity, which negatively correlates to the expression of nitric oxide synthase II (6–10). In contrast, “classically activated” M1 inflammatory macrophages enhance the production of free nitric oxide (NO) radicals, hereby eliminating intracellular parasites (11, 12). In human macrophages however, NO-mediated killing of *Leishmania* is still under debate, underlying the controversy of extrapolating immunological aspects from mouse to man (13–16). Nevertheless, antimicrobial peptides (AMPs), comprising defensins and cathelicidins, are key players in the human host's immune defense. In humans, only the cathelicidin antimicrobial protein hCAP18, encoded by the gene *CAMP*, has been identified. The *CAMP* gene product is cleaved to form the amphipathic, active LL37 peptide. LL37 can be found in various cell types, body fluids and tissues, such as the skin, where an increased production has been described to correlate with disease pathologies (17, 18). As a key molecule in host defense, LL37 exerts antimicrobial properties toward bacteria (*Staphylococcus* spp., *Pseudomonas* spp., *Mycobacteria* spp.), viruses, fungi, as well as parasites (19–26). Dos Santos et al. could demonstrate cathelicidin to exert anti-leishmanial activity in *L. donovani* infected macrophages, in line with data of Dos Santos et al. showing an IL-32/cathelicidin-mediated control of *L. braziliensis* in THP-1 cells (27). This AMP, LL37, able to create pores, hereby disrupting membranes. Although the exact mode of action is unknown, two models have been widely accepted being the “carpet” and “toroidal” model (17, 28). The toroidal model defines a pore architecture, formed by peptide channels, whereas the carpet model describes a more severe membrane perturbation, as seen for detergent-induced membrane destruction (29). In this study, we aimed to identify a role for the human cathelicidin during *Leishmania* infection. We could demonstrate *CAMP* to be upregulated in lesion material from Ethiopian individuals suffering from cutaneous Leishmaniasis. Using a human primary macrophage *in vitro* model, we identified *CAMP* to be upregulated specifically in pro-inflammatory macrophages and rLL37 was demonstrated to kill *Leishmania* in a dose dependent manner. By modulating the vitamin D pathway, we demonstrated *CAMP* expression to be upregulated, enhancing the macrophage's parasite killing capacity. In contrast, using a RNA interference (RNAi) approach in human primary macrophages targeting *CAMP* mRNA, the expression of hCAP18 was strongly reduced, enabling *Leishmania* parasites to survive better. In all, these data suggest an anti-parasitic activity of cathelicidin in a human primary *in vitro* cell model for cutaneous leishmaniasis and patient skin lesions.

## Results

### An Increased Expression of Cathelicidin in Skin Biopsies of African Patients With Cutaneous Leishmaniasis

**Figure 1:**
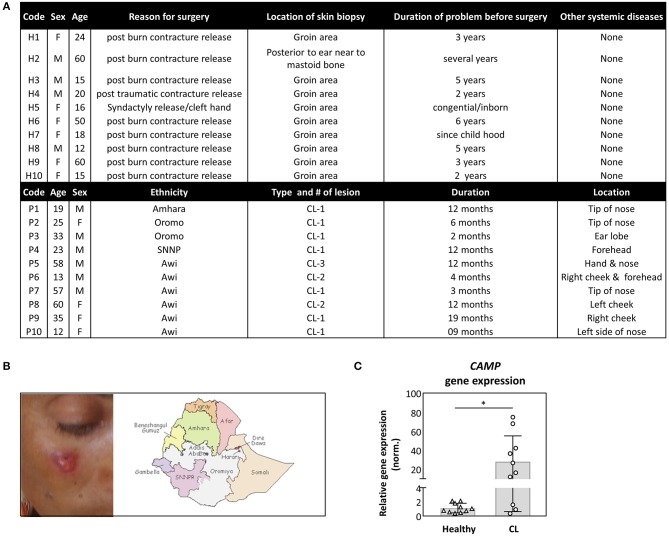
Increased expression of cathelicidin in skin biopsy of patients with cutaneous Leishmaniasis (CL). **(A,B)** Samples were collected from CL patients, with cutaneous lesions **(B)**, derived from people with a different ethnicity **(A)**. **(C)** Skin biopsies, from healthy (*n* = 10) or CL patients (*n* = 10), were collected from which *CAMP* gene expression was assessed by qRT-PCR. Relative gene expression was normalized against GAPDH and presented as mean ± SD. Statistical analysis (Mann–Whitney test) was used to compare groups using GraphPad statistical software (*p < 0.05).

In search for antimicrobial mechanisms in self-healing cutaneous Leishmaniasis (CL), we investigated the expression of human cathelicidin hCAP18. In Addis Ababa, Ethiopia, clinical samples from patients with CL and controls were collected and tested using RT-PCR. Patients varied in age, ethnicity, disease duration, and wound location, as depicted (Figures 1A,B). All patients were tested positive for the presence of *Leishmania aethiopica* by PCR. Interestingly, a significantly higher transcript abundance of the human cathelicidin hCAP18 was detected in skin biopsies of patients with CL, compared to control samples (Figure 1C).

### Dose Dependent Killing of *Leishmania* Parasites by Recombinant LL37

**Figure 2:**
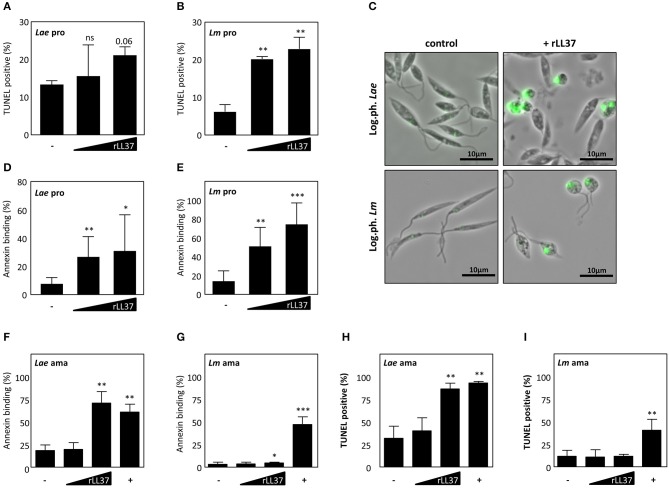
Dose dependent killing of *Leishmania* by human recombinant LL-37. Both logarithmic phase *Lm* and *Lae* promastigotes and axenic amastigotes were treated with different concentrations of rLL-37 or 25 μM staurosporine (+). After 24–72 h, DNA fragmentation was assessed by flow cytometry **(A,B)** for *Lae* and *Lm* promastigotes, respectively, and microscopy **(C)** and for *Lae* and *Lm* amastigotes **(H,I)**. Using flow cytometry Annexin binding was assessed by promastigotes **(D,E)** and axenic amastigotes **(F,G)** from *Lae* and *Lm*, respectively. Data are presented as mean ± SD of at least 3 independent experiments (paired *t*-test; *p < 0.05; **p < 0.01; ***p < 0.001; ns, not significant; pro, promastigotes; ama, amastigotes).

LL-37 and its precursor, hCAP18, are found in different tissue and cell types, playing an important role in innate immunity against diverse pathogens, e.g., *S. aureus, Mycobacterium tuberculosis, L. monocytogenes* (17). To define whether the hCAP18-derived peptide, LL37, is contributing to clearance of *Leishmania* parasites, we treated the promastigote and amastigote life stage of both *Leishmania major* (*Lm*) and *L. aethiopica* (*Lae*) with human recombinant LL37 (hrLL37). After treatment with hrLL37, DNA fragmentation and phosphatidylserine (PS) exposure, two hallmarks of apoptosis, were assessed. Treatment with increasing concentrations of hrLL37 resulted in a dose-dependent increase in TUNEL positivity for both *Lae* (15.5 ± 8.3%; 21.1 ± 2.3%) and *Lm* (20.2 ± 0.7%; 22.9 ± 3.1%), compared to untreated *Lae* (13.3 ± 1.2%) and *Lm* (6.24 ± 1.9%) promastigotes (Figures 2A,B). In addition, hrLL37 treatment induced a round-shaped morphology, as was described for apoptotic parasites (Figure 2C) (30). In line, hrLL37 treatment of promastigotes resulted in significant increase in AnnexinA5-binding parasites, in a dose-dependent manner, for *Lae* (27.1 ± 14.3%; 31.3 ± 26%) and *Lm* (51.5 ± 20.5%; 74.8 ± 23.2%), compared to untreated *Lae* (8.0 ± 4.5%) and *Lm* (14.7 ± 11.1%) (Figures 2D,E).

Treatment of *Lae* amastigotes also resulted in a significant increase in AnnexinA5 binding (71.3 ± 12.6%) and TUNEL positivity (87.0 ± 6.1%) compared to the untreated controls (18.7 ± 6.1%; 32.3 ± 13.3%) (Figures 2F–H). Interestingly, treatment of *Lm* amastigotes with rhLL37, resulted in only a minor but significant increase of AnnexinA5 binding (8.3 ± 3.0%), however TUNEL positivity (12.1 ± 2.1%) did not significantly increase, compared to the respective controls (3.3 ± 2.3%; 12.0 ± 6.6%) (Figures 2G–I). In all, we demonstrated hrLL37 to induce cell death characteristics, restricting parasites viability in a dose-dependent manner.

### Expression of Cathelicidin Is More Prominent in Pro-inflammatory Than in Anti-inflammatory Human Macrophages

**Figure 3:**
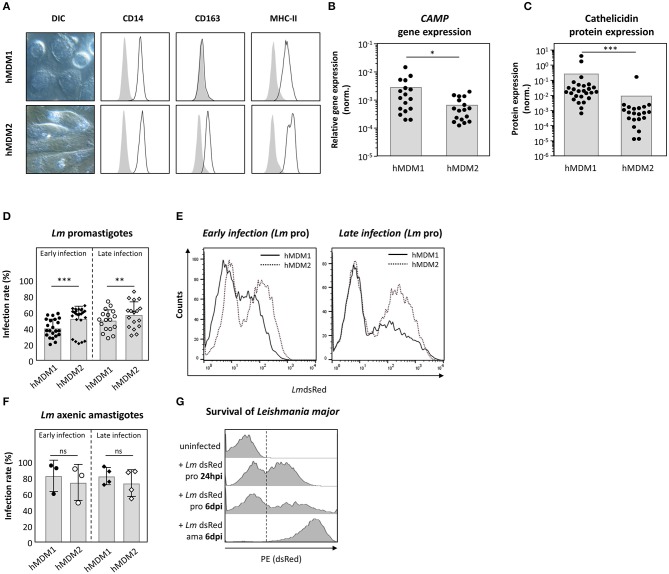
Expression of cathelicidin is more prominent in pro-inflammatory hMDM1 then in anti-inflammatory hMDM2. **(A)** Live cell imaging DIC micrographs of inflammatory hMDM1 and anti-inflammatory hMDM2, characterized by flow cytometry for CD14, CD163, and MHC-II surface expression (black line) in line with the isotype controls (gray). **(B,C)** *CAMP* gene expression (*n* = 17) **(B)** and cathelicidin protein expression (*n* = 21) **(C)** were assessed in hMDM1 and hMDM2 by qRT-PCR and western blot. *CAMP* expression was normalized against the house keeping gene *GAPHD*. Western blots were analyzed by densitometry (ImageJ analysis), normalizing cathelicidin against ß-actin protein expression. **(D–F)** Both hMDM1 and hMDM2 were infected with either transgenic *Lm* pro (*n* = 22–24) **(D,E)** or ama (*n* = 3–4) **(F)** (MOI of 10). After 3 h, extracellular parasites were removed by washing following incubation at 37°C, 5% CO_2_. After 24–48 hpi (early infection) or 6–7 dpi (late infection) infection rate was assessed by flow cytometry. **(G)** *Lm* dsRed pro transform into ama over time, indicated by an increased fluorescent intensity. Micrographs, histograms and data, presented as mean ± SD, are representative for at least 3 independent experiments (Wilcoxon matched-pairs signed rank test; *p < 0.05; **p < 0.01; ***p < 0.001; ns, not significant; pro, promastigotes; ama, amastigotes).

Human macrophages are key players during *Leishmania* infection. In a next step, the suitability of both human primary monocyte derived macrophages type 1 (hMDM1) and type 2 (hMDM2) as host for *Leishmania* parasites was assessed. From human blood, monocytes were isolated and differentiated using rhGM-CSF (10 ng/ml) or rhM-CSF (30 ng/ml), to generate hMDM1 or hMDM2, respectively. The hMDM1 were characterized by their fried-egg shaped morphology and CD14^+^MHCII^+^CD163^−^ phenotype (Figure 3A, upper lane). In contrast, anti-inflammatory hMDM2 have more elongated cell bodies and were phenotyped as CD14^+^MHCII^+^CD163^+^ (Figure 3A, lower lane). Interestingly, gene expression analysis demonstrated the gene *CAMP*, which encodes cathelicidin, to be significantly higher expressed in hMDM1, compared to hMDM2 (Figure 3B). Also a significant elevated cathelicidin protein amount was present in hMDM1 (0.25 ± 0.85) compared to hMDM2 (0.01 ± 0.04) (Figure 3C). In a next step, both hMDM phenotypes were infected with transgenic *Lm* promastigotes or axenic amastigotes, after which parasite infection rate (*Lm*dsRed^+^ hMDM) was assessed. Of note, the dsRed protein is constitutively expressed, in viable *Leishmania* parasites, as described previously (31). At early (24–48 hpi) and late time points (6–7 dpi) after promastigote infection, a significantly higher infection rate was observed in hMDM2 (52.7 ± 15.6%; 57.4 ± 16.4%), compared to hMDM1 (40.8 ± 11.5%; 50.4 ± 13.4%) (Figures 3D,E). Infection with amastigotes resulted in a high infection rate, which however did not differ significantly between early and late time points in hMDM1 (83.2 ± 19.5%; 75.0 ± 22.4%) and hMDM2 (82.9 ± 10.8%; 74.2 ± 16.6%), respectively (Figure 3F). Of note, the transgenic *Lm* promastigotes transformed into amastigotes *in vitro*, as the expression of dsRed increased (increase in mean fluorescent intensity, MFI) (Figure 3G). Altogether, pro-inflammatory human macrophages were demonstrated to express cathelicidin to a higher extent, which may contribute to an impaired parasite survival.

### Vitamin-D Derivatives Induce Cathelicidin-Mediated-*Leishmania* Restriction in Human Primary Macrophages

**Figure 4:**
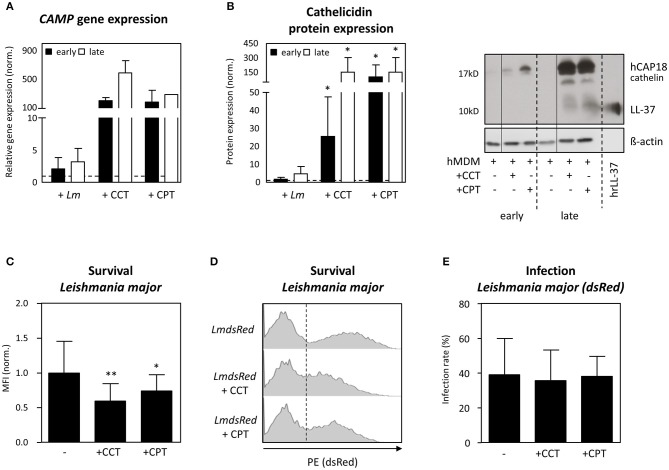
Vitamin-D derivatives induce LL37 mediated Leishmania restriction in human primary macrophages. hMDM-1 were generated and treated with calcitriol (CCT) or calcipotriol (CPT) for 24 h, or left untreated. Subsequently, hMDM-1 were infected with transgenic dsRed expressing *Lm* or *Lae* (MOI10). After 3 h, extracellular parasites were removed by washing, following incubation at 37°C, 5% CO_2_. After 24 h (early; black bars) or 6 d (late; white bars) *CAMP* gene (*n* = 1–5) **(A)** and cathelicidin protein expression (*n* = 5–7) **(B)** were by qRT-PCR and western blot analysis. *CAMP* expression was normalized against the house keeping gene *GAPHD*. Western blots were analyzed by densitometry (ImageJ analysis), normalizing cathelicidin against ß-actin protein expression. Gene and protein expression were normalized to the untreated control, which was set to 1 (dashed line). In addition, parasite survival **(C,D)** and infection rates of *Lm* dsRed **(E)**, analyzed by the mean fluorescent intensity (MFI) of the dsRed protein, was assessed by flow cytometry. Western blots, FACS histograms and data, presented as mean ± SD, are representative for at least 3 independent experiments (Wilcoxon matched-pairs signed rank test; *p < 0.05; **p < 0.01).

To investigate the role of cathelicidin in *Leishmania* restriction further, we increased expression of cathelicidin using Vitamin D derivatives. Upon activating the Vitamin-D pathway in hMDM1, using 1α, 25-dihydroxyvitamin D3 (calcitriol, CCT, 100 nM) and calcipotriol (CPT, 100 nM), a synthetic VitD3 analog, we assessed *Leishmania* parasite survival (32). Already 24 h post infection (early), assessment of *CAMP* gene expression showed *Lm*-infected hMDM1 to have a slightly increased *CAMP* expression (2.1 ± 1.8-fold), compared to the uninfected control (normalized to 1; dashed line) (Figure 4A). Treatment with CCT and CPT resulted in a 199- (± 41.1) fold and 181- (± 164) fold increase of *CAMP*. After 6 days (late), an even higher expression of *CAMP* was detectable after treatment with CCT (587 ± 173-fold), CPT (282-fold) or during *Lm* infection (3.2 ± 2.1-fold) (Figure 4A). In line, protein expression was assessed by Western blot, demonstrating an increased cathelicidin protein expression during *Leishmania* infection (early: 1.8 ± 1.0-fold; late: 4.8 ± 4.0-fold). Furthermore, both CCT and CPT induced a significant increase in cathelicidin protein expression at early (25.5 ± 22.2-fold; 101.9 ± 125.4-fold) and at late time points (152.5 ± 149.6-fold; 101.9 ± 125.4-fold), respectively (Figure 4B). In a next step, hMDM1 were pretreated with CCT or CPT, followed by infection with transgenic dsRed-expressing *L. major* or *L. aethiopica*. Using flow cytometry, infection rates and parasite survival, as mean fluorescent intensity (MFI), were assessed. Remarkably, treatment with CCT and CPT significantly reduced *L. major* parasite survival (0.60 ± 0.25; 0.74 ± 0.23) compared to the control (1.00 ± 0.46) (Figures 4C,D). However, the percentage of hMDM1 infected cells, indicated as infection rate, did not change significantly (Figure 4E). Regarding *L. aethiopica*, similar findings were acquired (data not shown). These data demonstrate that targeting the Vitamin D pathway strongly compromises *Leishmania* parasite survival, as cathelicidin is strongly upregulated.

### Reduction of Endogenous Cathelicidin in Human Primary Macrophages Promotes *Leishmania* Infection

**Figure 5:**
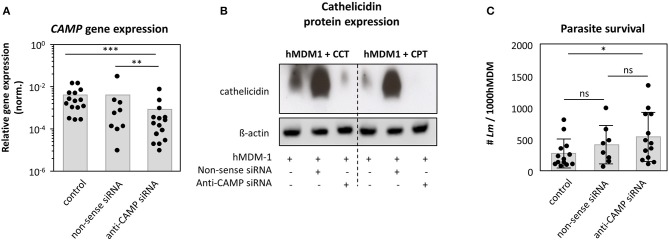
Knockdown of CAMP in human primary macrophages promotes *Leishmania* infection. Human pro-inflammatory macrophages, hMDM-1, were treated with non-sense or anti-CAMP siRNA for 7 h. After 2 d cells were harvested and *CAMP* gene expression and protein expression was assessed by qRT-PCR (*n* = 9–15) **(A)** and western blot upon inducing cathelicidin expression using calcitriol (CCT) or calcipotriol (CPT) **(B)**. *CAMP* expression was normalized against the house keeping gene *GAPHD*. Western blots were analyzed by densitometry (ImageJ analysis), normalizing cathelicidin against ß-actin protein expression. **(C)** Subsequently, cells were infected with *Lm* (MOI10) for 48 h followed by an end-point titration assay (see methods) to assess parasite survival (*n* = 8–13). Data are presented as mean ± SD and are representative for at least 3 independent experiments (Wilcoxon matched-pairs signed rank test; *p < 0.05; **p < 0.01; ***p < 0.001; ns, not significant).

The human cathelicidin is expressed by monocytes, macrophages as well as neutrophils (17, 33). We already demonstrated (i) cathelicidin to be higher expressed in skin biopsies of patients with the self-healing cutaneous leishmaniasis compared to healthy controls, (ii) hrLL37 to facilitate apoptosis among promastigotes and amastigotes and (iii) hMDM1 to express cathelicidin to a higher extent comparted to hMDM2. To demonstrate a role for the intracellular, endogenous cathelicidin of macrophages in the elimination of *Leishmania* parasites, knockdown (KD) experiments were performed. Using an RNAi approach, we could significantly reduce *CAMP* gene expression (0.15 ± 0.13), compared to the control (1.0 ± 0.0) and non-sense siRNA control (1.04 ± 0.74) (Figure 5A). We were not able to show a clear cathelicidin protein decrease in siRNA treated cells by western blot (data not shown), as cathelicidin is expressed at low levels under steady state conditions. Therefore, we assessed cathelicidin protein expression in knockdown cells by triggering the Vitamin-D pathway first with CPT and CCT, including β-actin as loading control, demonstrating a strong reduction of cathelicidin protein amount, upon CCT and CPT treatment, in the KD cells compared to the control cells (Figure 5B). Next, KD and control cells were infected with *Leishmania* promastigotes, after which intracellular survival was investigated using an end-point titration assay. We could demonstrate that the number of viable *Leishmania* in control (284 ± 228 *Lm*) and non-sense siRNA treated cells (421 ± 303 *Lm*) did not significantly differ. However, KD of *CAMP* resulted in a higher parasite survival (544 ± 388 *Lm*) (Figure 5C), although the level of significance was not reached. In all, these data show cathelicidin to play a role in the restriction of *Leishmania* promastigote survival.

## Discussion

In the present study, we were able to define a role for the human cathelicidin in human leishmaniasis, based on data from clinical samples. In addition, a human primary *in vitro* cell model was designed to better mimic the *in vivo* interaction between *Leishmania* parasites and their host cell, the human macrophage. The two phenotypes of macrophages were demonstrated to interact differently with *Leishmania* parasites, as in anti-inflammatory macrophages are more susceptible compared to pro-inflammatory human macrophages. Furthermore, the latter pro-inflammatory phenotype expressed the cathelicidin *CAMP* gene transcript and protein more strongly, which we demonstrated to contribute in controlling *Leishmania* infection.

### Cathelicidin's Antimicrobial Activity

Cathelicidins have already been described to play a role during infection with e.g., *M. tuberculosis, Candida albicans*, and *Cryptosporidum parvum* (34). For *Leishmania* infection animal studies, numerous reports are present. Ramos et al. demonstrated a reduced disease spreading in *Leishmania mexicana* infected BALB/c mice, which were supplemented with calcitriol (35). Ehrchen et al. reported vitamin D receptor KO mice to be more susceptible to infection then control mice (36). In line, CAMP was demonstrated to be crucial for the local control of cutaneous lesion development and parasite growth, using CAMP KO mice (25). Furthermore, progression of visceral Leishmaniasis was demonstrated to be associated with vitamin D deficiency in dogs (37). Few data however, evaluate the effect of cathelicidin and/or the vitamin D pathway in human patients and/or a human cell model. Das et al. could show cathelicidin to augment anti-leishmanial macrophage activating properties of Amphotericin B (38). In line, we identified a strong upregulation of the human CAMP mRNA transcript in clinical samples from African patients with cutaneous Leishmaniasis, suggesting cathelicidin to play a role in human CL *in vivo*.

### Cathelicidin-Induced Apoptotic Death of *Leishmania*

We could demonstrate the human cathelicidin to induce an apoptosis-like phenotype in *Leishmania* parasites, in a dose dependent manner in both *L. major* and *L. aethiopica* promastigotes as well as in *L. aethiopica* amastigotes. Although the underlying mode of action remains elusive, rLL37 was demonstrated to induce phosphatidylserine exposure, a round shaped cell morphology and DNA fragmentation, all characteristics of apoptosis (39). Presumably, the amphipathic α-helical peptide LL37 interacts with the negatively charged phospholipids within the parasitic membrane by electrostatic forces, as described for the carpet and toroidal-pore model (17). Surprisingly, recombinant LL37 did not exert apoptosis-inducing effect on the *L. major* amastigote life stage, when looking at TUNEL positivity and DNA degradation. Of note, Kulkarni et al. could show antimicrobial peptides to differently induce parasitic cell death, by means of non-apoptotic (class I) or apoptotic (class II) mediated killing (26). One could speculate these mechanisms to be also applicable in our model, which will be the focus of future research.

Amastigotes also differ in their surface charge compared to promastigotes, as Pimenta et al. could show transformation of *Leishmania mexicana amazonensis* promastigotes to amastigotes to be associated with a shift in the electrophoretic mobility (40). Of general acceptance, is the fact that cationic antimicrobial peptides strongly bind negatively charged phospholipid moieties. Due to the different surface charge between *Leishmania* life stages, we speculate LL37 to only bind *Lm* promastigotes resulting in killing, whereas LL37 to be ineffective in binding *Lm* amastigotes. Overall, our data indicate rLL37 to induce cell death in *Leishmania* promastigotes.

### Cathelicidin in Mammalian Innate Immune Defense

Cathelicidins have gained increasing attention, as being an important mediator during innate immunity. Although cathelicidins are primarily present in human neutrophils, also keratinocytes, monocytes and macrophages harbor this antimicrobial peptide (17, 33, 41–43). These cells may indicate where the cathelicidin is originating from upon *Leishmania* infection. Whether macrophages and keratinocytes exert synergistic effects with regard to cathelicidin production and *Leishmania* elimination is yet to be defined. Focusing on human primary macrophages, we could demonstrate different macrophage phenotypes to express cathelicidin to a different extent. The human cathelicidin is more abundant in pro-inflammatory macrophages, which may not be surprising as it drives macrophages polarization to a pro-inflammatory phenotype (44). The anti-inflammatory macrophages were more susceptible for infection, a finding in agreement with previous data and studies (45, 46). To define an active role for cathelicidin during *Leishmania* infection, we modulated its expression. In concordance with previous studies, we could show *CAMP* expression to be highly enhanced upon activating the vitamin D pathway, using calcitriol or calcipotriol (47–50). Interestingly, the intracellular survival of *Leishmania* parasites was significantly impaired. The group of Agerberth could show LL37 induced expression to be associated with the control of *M. tuberculosis* in human macrophages (51). Furthermore, phenyl butyrate/vitamin D3 treatment, induced LL37-mediated elimination of *M. tuberculosis* by macrophages, strengthening the data of the Modlin's group, showing cathelicidin to be required for the 1,25D(3)-triggered antimicrobial activity against intracellular *M. tuberculosis* (52, 53). In all, triggering the vitamin D pathway in human macrophages, hereby inducing cathelicidin expression, restricts *Leishmania* survival.

### Cathelicidin Contributes to a Reduced Parasite Survival

Vitamin D derivatives induce expression of diverse immune modulators, such as cathelicidin, IL-1ß, etc. (54). To target the *CAMP* gene more specifically, a RNA interference (RNAi) approach was chosen. Our data showed *Leishmania* parasite survival to be enhanced. Of note, no significant difference was observed between non-target and anti-CAMP siRNA treatment. One should keep in mind, that all human macrophages were derived from human blood donors, which may differ in gender, immune status, etc., having an impact on host pathogen interactions (55). Furthermore, also transfection as a treatment, may result in RNAi associated immune stimulation through activation of IFN signaling cascades (31). Both aspects, might “bias” our results, with regard to the comparison to the untreated control, as type I interferons have been demonstrated to increase superoxide dismutase (SOD) expression in macrophages, favoring parasite survival (56). Besides the restrictions of the employed methodology, a stronger tendency toward parasite survival, upon *CAMP* RNAi was observed, suggesting cathelicidin to contribute in restricting *Leishmania* parasite survival. Indeed, McGwire's group could show the corresponding murine cathelicidin (*CRAMP*) to control *Leishmania* parasite infection in a mouse infection model (25). Knockout mice for *CRAMP* were reported to develop exacerbated lesions combined with a higher parasites distribution upon *L. major* infection as compared to wild type mice (25). Of note, Gombart et al. showed a vitamin D response element (VDRE) to be conserved in the *CAMP* promotor of primates. The absence of the VDRE region in the genomes of mouse, rat and canine makes the expression of *CRAMP* not tunable by the vitamin D pathway (57). In humans, the great potential of cathelicidin is also highlighted in other disease pathologies. A deficiency of cathelicidin may impede the outcome of inflammation in the lungs of patients with severe sarcoidosis (58). Furthermore, Searing et al. propose an increased production of LL37 to prevent patients with atopic dermatitis from herpes infection (59).

## Conclusion

In the current study, we revealed the *CAMP* transcript to be strongly upregulated in skin lesion material from cutaneous leishmaniasis patients. Using an *in vitro* model, we demonstrated pro-inflammatory human macrophages to be able to control *Leishmania* infection more efficiently compared to anti-inflammatory macrophages, to which cathelicidin expression is contributing. In addition to the NO-based anti-leishmanial mouse effector mechanism, we propose that vitamin D-inducible cathelicidin expression in combination with GM-CSF polarized macrophages to be a unique mechanism, which contributes to the restriction of *Leishmania* in human macrophages.

## Materials and Methods

### Leishmania Strains

*L. major* (*Lm*, MHOM_IL_81_FEBNI), *L. aethiopica* (*Lae*, MHOM/ET/72/L100 Z14), and the transgenic *Lm* dsRed (construct pSSUint-DsRed was a kind gift from Dr. Toni Aebischer, Robert Koch Institute, Berlin, Germany) and *Lae* dsRed promastigotes were cultured at 27°C in biphasic Novy-Nicolle-McNeal blood agar medium as described (60). Of note, all viable promastigotes express the transgenic dsRed protein. Upon transformation into the amastigote life stage, a 1 log-scale higher dsRed fluorescence is present, due to the high-level expression in the amastigote life stage (31). Logarithmic-phase or stationary-phase promastigotes were obtained after 2 (log-phase) or 7 (stat-phase) days of culture, respectively. *Lm* and *Lae* axenic amastigotes were generated by incubating log-phase promastigotes in pH 5.5 at 33°C and isolated using a discontinuous Histopaque® 1119 (Sigma Aldrich, Germany) density gradient as described (61).

### Assessing Apoptosis

To assess apoptosis, *Lm* and *Lae* promastigotes, which resided in a logarithmic growth phase, were treated with 30 and 60 ng/ml hrLL37 (PeptaNova GmbH, Sandhausen, Germany) for 72 h. *Leishmania* axenic amastigotes were treated with 10 and 100 ng/ml hrLL37 or 25 μM staurosporine for 24 h. Subsequently, DNA fragmentation was assessed by flow cytometry or immunofluorescence imaging using an *in situ* cell-death detection kit, based on terminal deoxynucleotidyl transferase dUTP nick end labeling (TUNEL), as described (30). Exposure of phosphatidylserine (PS) was assessed by AnnexinA5 binding using flow cytometry.

### Ethics Approval and Consent to Participate

The study was approved by National Ethical Clearance Committee at Federal Democratic Republic of Ethiopia Ministry of Science and Technology with ethical approval No. 310/227/2007, approved on 30/05/2011. The ethical approval was renewed by the National Ethical Clearance Committee at Federal Democratic Republic of Ethiopia Ministry of Science and Technology on 26/03/2016 with Ref. number 3.10/003/2015. Written informed consent was obtained from study participants.

### Sample Collection

Clinically suspected CL patients, who visited the Ankesha and Kela health centers consented to participate in the study, were clinically examined for CL. Ankesha and Kela health centers are found in the leishmaniasis endemic regions in East Gojam Zones of Amhara region and in Gurage zone of Southern regional state of Ethiopia, respectively. Patients, diagnosed for active CL, were recruited to this study prior to treatment. Diagnosis was confirmed by microscopy or culture from skin lesion scraping. After the skin lesion was cleaned, the boarder of lesion was collected for microscopy analysis. Healthy controls were recruited from patients admitted for minor surgery ALERT hospital. All study participants were seronegative for HIV. Skin biopsies from CL patients were taken from the border site of the lesion, using a disposable punch (3 mm in diameter). Local anesthesia with 2% lidocaine was applied. Control skin biopsies were obtained from the leftover samples taken for skin graft of selected individuals (without infection or immunological disorder) visiting the ALERT hospital surgery department.

In addition, from skin samples *Leishmania* promastigotes were cultivated. DNA was extracted from culture and biopsy samples using QIAamp® DNA Mini Kit according to manufacturer's procedure. PCR amplification was performed with 100 ng template and the HotStarTaq Plus Master Mix Kit (Qiagen, Hilden, Germany) using the primers *Lae* species-specific primers V5F 5′-GGTGATGTGCCCGAGTGCA-3′ and V10R 5′-CGTGCACATCAGCACATGGG-3′.

### Generation of Human Monocyte-Derived Macrophages

Human peripheral mononuclear cells (PBMCs) were isolated from buffy coats (DRK-Blutspendedienst Hessen GmbH) by passage over a Leukocyte Separation Medium gradient as described previously (30). Monocytes, obtained by plastic adherence or CD14 selection were incubated either with 10 ng/ml rhGM-CSF (Leukine® Sanofi-Aventis, Bridgewater, US) or 30 ng/ml rhM-CSF (R&D Systems, Abingdon, UK) for a period of 5 to 7 d at 37°C, 5% CO_2_ to generate hMDM1 or hMDM2, respectively. Cells were generated in 6 w plates or in 25 cm^2^ culture flasks and were detached by cooling cells down on ice, following detachment with a cell scraper. Experimental data, conducted with monocytes obtained from human donors, are depicted as dot plots, in which each dot presents data from a single donor.

### Infection of hMDM With *Leishmania* Parasites

HMDM were harvested and transferred into 1.5 ml micro-centrifuge tubes, to which cells do not attach. The cells were co-incubated with stationary phase *Lm* or *Lae* promastigotes or axenic amastigotes at a MOI ratio of 1:10 in RPMI 1640 supplemented with 10% heat inactivated fetal calf serum, 50 μM β-mercaptoethanol (all from Sigma Aldrich), 2 mM L-glutamine, 100 U/ml penicillin and 100 μg/ml streptomycin, 10 mM HEPES (all from Biochrom) for 3 h at 37°C in a humidified atmosphere in a CO_2_ incubator. Extracellular parasites were removed by centrifugation and washing the cells. During infection experiments, cathelicidin expression was induced by incubation of hMDM with 100 nM calcitriol or calcipotriol for 24 h. For transgenic *Lm*dsRed flow cytometry was used to analyze infection rates. These transgenic parasites can also be used as a model to follow the parasite propagation, which is based on the development and replication of amastigotes. The *Lm*dsRed promastigotes increase their fluorescence intensity when transforming into amastigotes, which enables the quantification of the parasite propagation by measuring the dsRed mean fluorescence intensity using FACS (62, 63).

### Flow Cytometry

- FACS analysis of *Leishmania* parasites was performed as described Wenzel et al. (61). Apoptosis, among parasites, was assessed by staining with AnnexinA5-Alexa Fluor 647 using a Ca^2+^ rich buffer.
- For phenotyping hMDM by flow cytometry, hMDM were washed in FACS-Buffer (PBS supplemented with 1% FCS, 1% human serum and 1% BSA) and incubated with anti-CD14-FITC (1:100, IgG2b), anti-CD163-PE (1:50, IgG1, GHI/61), or anti-MHC II-PerCP (1:100, IgG2a, L243) for 30 min on ice in the dark. Corresponding isotype controls were used in the same dilution (all antibodies and isotype controls were from BD Pharmingen, Heidelberg, Germany). The cells were washed in FACS-Buffer and analyzed by flow cytometry.
- To assess parasite survival, Leishmania (dsRed^+^) infected hMDM were washed in FACS-buffer and infection rate (% of dsRed^+^ hMDM) and the parasite load (mean fluorescent intensity) were assessed by flow cytometry. Upon analyzing, at least 10,000 events (human cells) or 20,000 (parasites) were recorded using a BD LSR II flow cytometer (BD Bioscience, Heidelberg, Germany). Data were analyzed by BD FACS Diva or FlowJo software (Treestar).

### Transfection of Primary Human Cells With siRNA

CD14 selected monocytes were differentiated into hMDM1 by addition of hrGM-CSF over a period of 6 days. On day 3, the medium was refreshed with new growth factors. At day 6, cells were washed with RPMI, without supplements, and 1 ml prewarmed RPMI was given to the hMDM. For transfection 80 pmole of 20 μM siRNA (LL-37-siRNA: ON-TARGET plus SMART pool Human CAMP (LL-37) from Thermo Scientific Dharmacon, Bonn, Germany; nonsense siRNA: Stealth RNAi siRNA Negative Control from Invitrogen, Darmstadt, Germany) were mixed with 20 μl of Stemfect Buffer and 4.6 μl of Stemfect Reagent were mixed with 20 μl Stemfect Buffer (Stemfect RNA Transfection kit from Stemgent, San Diego, USA). Within 5 min, both compounds were mixed and subsequently incubated for 20 min at room temperature. The transfection mixture was added to the cells for 7 h at 37°C. Cells were subsequently washed and further incubated 2 days at 37°C in Complete Medium before harvesting.

### RNA Isolation, Reverse-Transcription PCR, and qRT-PCR

RNA extraction and reverse-transcription, of either human primary cells or skin tissue, were performed using the RNeasy Plus Mini kit (Qiagen, Hilden, Germany) and the ImProm-II Reverse Transcription system (Promega, Mannheim, Germany), respectively, according to the manufacturers' instructions. Differential gene expression in primary macrophages was analyzed by quantitative real-time PCR using the LightCycler® 2.0 instrument (Roche Diagnostics GmbH, Mannheim, Germany) and LightCycler® FastStart DNA MasterPLUS SYBR Green I kit (Roche Applied Science, Mannheim, Germany) according to the manufacturer's instructions. Differential gene expression in skin tissue was analyzed by quantitative real-time PCR using the Rotor Gene-3000 system and Rotor-Gene SYBR Green I kit (Qiagen) according to the manufacturer's instructions. The LL-37 gene (5′-GGA CCC AGA CAC GCC AAA-3′; 3′-GCA CAC TGT CTC CTT CAC TGT GA-5′) expression was normalized to the housekeeping gene GAPDH (5′-GAG TCA ACG GAT TTG GTC GT-3′; 3′-TTG ATT TTG GAG GGA TCT CG-5′) using the ΔΔCT–method.

### Western Blot

A total number of 0.5 × 10^6^ hMDM were lysed in Lämmli-Buffer (A. bidest supplemented with 0.7 M Glycerol, 1.7% SDS, 0.1 M DTT and 30 μM Bromphenol blue), denaturated at 95°C for 10 min and loaded onto a 15% SDS-polyacrylamide gel. The separated proteins were blotted onto a nitrocellulose transfer membrane at 145 mA constant voltage for 1 h. The membrane was blocked with WB-Block-Solution (GE Healthcare, Buckinghamshire, United Kingdom) washed with WB-Wash-Buffer (A. bidest supplemented with 0.5% Tween, 0.14 M NaCl, 10 mM Tris, 1 mM NaN3; pH 8) and subsequently incubated with an anti-LL37 primary antibody (kindly provided by Prof. B. Agerberth) overnight at 4°C. After extensive washing, the membrane was incubated with a HRP-conjugated secondary antibody (1:1000, from Cell Signaling, Danvers, USA) for 1 h at room temperature. The membrane was washed once more and the protein bands were detected using an ECL substrate (GE Healthcare, Buckinghamshire, United Kingdom). Using ImageJ, densitometry analysis was performed to quantify the intensities of the protein bands.

### End-Point Titration Assay

The amount of viable intracellular parasites inside hMDM1 (control, non-sense siRNA or anti-CAMP treated) was determined. After 3 h of infection, MOI10, cells were washed to remove extracellular parasites. After 48 h of infection, the end-point titration assay was carried out. Therefore, cell scraper detached hMDM1 were counted and 2000 hMDM1 were seeded, in quadruplicates, in a 96 w plate containing biphasic Novy-Nicolle-McNeal blood agar medium. Wells were serial diluted (factor 1.5) for 24 times. Plates were incubated for 7–10 days at 27°C. By microscopical analysis, plates were analyzed to assess at which dilution growth was seen. Based on the dilution factor and the amount of hMDM1 that were seeded in the first well, the amount of parasites per 1,000 hMDM1 was calculated (63). The formula applied to calculate the amount of parasites per 1,000 macrophages is 1.5^x^/2 were x is the dilution step in which still parasite growth was observed.

### Statistical Analysis

Numerical data are presented as the mean ± standard deviation (SD). For statistical analysis, data were tested for their normal distribution, using the D'Agostino and Pearson omnibus normality test. If passed, statistical analysis was determined by a paired Student *t*-test. If data were not normally distributed or in case to few biological replicates were present to test normal distribution, a non-parametric test (Mann–Whitney test or Wilcoxon matched-pairs signed rank test) was used. The software Graph-Pad Prism version 4 was used, by which ^*^ indicates statistically difference at *p* < 0.05, ^**^p < 0.01, ^***^p < 0.001.

## Data Availability Statement

All datasets generated for this study are included in the article/supplementary material.

## Ethics Statement

The study was approved by National Ethical Clearance Committee at Federal Democratic Republic of Ethiopia Ministry of Science and Technology with ethical approval No. 310/227/2007, approved on 30/05/2011. The ethical approval was renewed by the National Ethical Clearance Committee at Federal Democratic Republic of Ethiopia Ministry of Science and Technology on 26/03/2016 with Ref. number 3.10/003/2015. Written informed consent was obtained from study participants.

## Author Contributions

GZ, PC, EB, BW, UW, MC, and MA helped with substantial contributions to the conception or design of the work, or the acquisition, analysis or interpretation of data for the work. GZ, PC, BA, RK, NR, and UR helped drafting the work or revising it critically for important intellectual content.

### Conflict of Interest

The authors declare that the research was conducted in the absence of any commercial or financial relationships that could be construed as a potential conflict of interest.
